# 781. “Rashional” Infectious Diseases Training: A “Dermatology for Infectious Diseases Fellows” Pilot Curriculum Evaluation

**DOI:** 10.1093/ofid/ofad500.842

**Published:** 2023-11-27

**Authors:** Danica Rockney, Michael T Melia

**Affiliations:** University of Michigan, Ann Arbor, Michigan; Johns Hopkins University, Baltimore, MD

## Abstract

**Background:**

Rashes are common among patients with infections. A prior survey of United States adult infectious disease (ID) fellowship program directors showed that most respondents (55/90; 61%) do not have a formal dermatology curriculum for their fellows and 76% (41/54) were interested in incorporating an externally produced dermatology curriculum if made available to them. To address this education gap, a “Dermatology for the ID Fellow” curriculum was created.

**Methods:**

During the 2022-2023 academic year, a “Dermatology for the ID Fellow” curriculum was piloted with the 11 fellows in the Johns Hopkins adult ID fellowship program. The curriculum included two lectures, multiple asynchronous board-style questions, and three knowledge assessments (Figure 1). Curricular objectives were evaluated by pre-, mid-, and post-curriculum knowledge assessments. Descriptive data analysis was performed. This study was IRB exempt.

**Figure:**
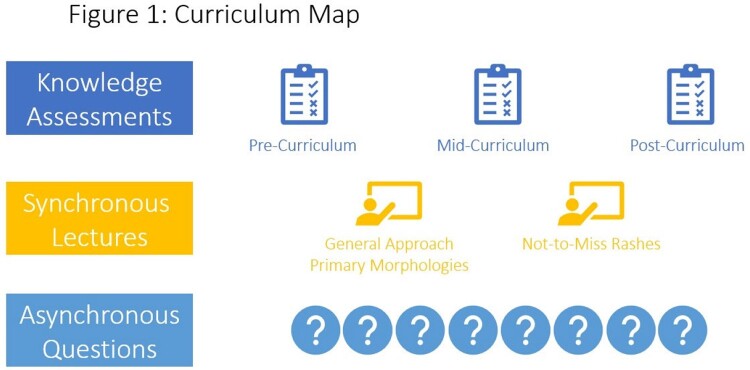

**Results:**

The response rates for the pre-, mid-, and post-curriculum knowledge assessments were 91%, 64%, and 55% respectively. The response rate of asynchronous questions averaged 63% across all participating fellows (range 13%-100%). Most fellows correctly identified primary rash morphologies by verbal description (88% correct) and photographs (96% correct). At the end of the curriculum, 50% (4/8) of the fellows ranked their comfort level in approaching cutaneous manifestations of infections as higher than before the curriculum, with 38% (3/8) staying the same. More fellows used a systematic approach to unknown rashes after the curriculum (83%; 5/6) as compared to before the curriculum (30%, 3/10) (Figure 2). There was no improvement in fellows' ability to determine a differential diagnosis based on rash appearance. Overall, 100% of the fellows felt like the lectures presented new and important knowledge.

**Figure:**
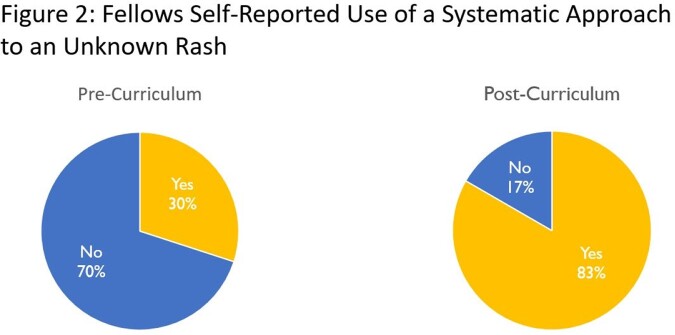

**Conclusion:**

Incorporation of a “Dermatology for ID Fellows” curriculum was viewed favorably. The curriculum increased the use of a systematic approach to a rash and helped some fellows improve comfort level in approaching a rash. Data from this pilot curriculum will inform subsequent iterations that may help increase the ability to develop infectious differentials based on rash appearance, addressing an important knowledge gap in ID fellowship training.

**Disclosures:**

**All Authors**: No reported disclosures

